# Metastatic Squamous Cell Carcinoma of the Anus: Time for a Shift in the Treatment Paradigm?

**DOI:** 10.5402/2012/756591

**Published:** 2012-05-06

**Authors:** Alice Dewdney, Sheela Rao

**Affiliations:** Department of Medicine, Royal Marsden Hospital, London and Surrey, UK

## Abstract

Anal cancers are rare tumours; however, the incidence is increasing in both men and women. Changing trends in sexual behaviour, smoking, and infection with the human papillomavirus are thought to be responsible for the increase. Patients with metastatic disease have a poor prognosis, with 5-year median overall survival rates of 10% in men and 20% in women. The standard systemic treatment of metastatic disease remains cisplatin and 5-fluorouracil, and aside from several non-randomised small phase II trials there has been no real progress over the past two decades. Based on the efficacy of cetuximab in squamous cell carcinomas from other primary sites, there appears to be clinical rationale for evaluation of anti-epidermal growth factor inhibitors in anal squamous cell carcinoma. In order to facilitate research and implement more effective treatment strategies international collaboration in clinical trials incorporating tissue collection for biomarkers is essential.

## 1. Introduction

Anal cancers are rare tumours; in the UK there are approximately 900–1000 cases diagnosed per year, and in the USA they account for 1-2% of all gastrointestinal cancers [1]. However, the incidence is increasing in certain populations with high-risk behaviour and in those with the human immunodeficiency virus (HIV) [2, 3]. One of the most well-known risk factors is the human papillomavirus (HPV), particularly HPV type 16, which is present in approximately 80% of patients diagnosed with anal cancer [4]. Other risk factors include smoking, increased number of sexual partners, sexually transmitted infections, a history of vaginal or cervical malignancy, other conditions associated with lowered immunity, for example, transplant recipients and those with anal inflammatory conditions. Eighty percent of anal cancers are squamous cell carcinomas (SCCs); other rarer types of histology include adenocarcinoma, basal cell carcinoma, and melanoma [5].

The majority of cases present with early-stage localized disease. In this setting combined modality treatment with chemoradiation remains the standard of care allowing sphincter sparing whilst reserving surgery for those with persistent or recurrent disease following treatment [6]. Based on the available data a radiation dose of at least 45–50 Gy in combination with MMC and 5FU is currently the most commonly employed regimen [7–10].

Early-stage disease (T1/T2 N0) is associated with a good prognosis [11]. However, the five-year overall survival for those with more locally advanced disease ranges from 40–80% and 10 to 20% of patients will develop distant metastases following combined modality treatment [10, 12]. The prognosis for patients with distant metastases is generally poor, although documented median survival rates vary from 8 to 34 months. The SEER database reports 5-year survival rates of 10% in men and 20% in women between 1973 and 2000 [2, 11].

This paper reviews the existing literature regarding systemic treatment of patients with metastatic squamous cell carcinoma of the anus and explores potential future directions.

## 2. Trials of Systemic Chemotherapy

It is striking that in anal cancer only six randomized trials have been performed over the last three decades. In the metastatic setting the available evidence is based on small phase II trials (Table 1), retrospective series, and case reports (Table 2), and there have been no prospective phase III studies or meta-analyses.

The most plausible explanation is simply the relative rarity of the disease and the small number of patients treated at each centre. Unfortunately anal cancer still suffers from a social stigma with an “embarrassing location” and the association with sexually transmitted infections and HIV. There has certainly not been the same degree of public interest and support for anal cancer as demonstrated in other solid tumours, for example, breast or testicular cancer.

Systemic chemotherapy remains the mainstay of treatment for patient with metastatic disease. The choice of chemotherapy is influenced by any previous treatment the patient received for early disease, the disease-free interval, and the patient's performance status and wishes. The National Comprehensive Cancer Network (NCCN) Guidelines currently recommend cisplatin and 5FU chemotherapy as first-line treatment of metastatic squamous cell carcinoma (SCC) [21]. This is largely based on a study of 19 patients treated with cisplatin 100 mg/m^2^ and infusional 5FU 1 gm/m^2^/day over 5 days with a 66% response rate; there was 1 complete response and 11 partial responses in addition to 4 patients with stable disease. The actuarial survival was 62.2% at 1 year and 32.2% at 5 years, and the median survival was 34.5 months [22]. There have also been a number of case reports demonstrating a benefit with the cisplatin/5FU combination [23–25].

Recently the oral fluoropyrimidine capecitabine has been substituted for 5FU in a number of solid tumours, and, extrapolating from this data, many centres now use capecitabine with cisplatin rather than 5FU [26]. This has a number of advantages for patients, including ease of administration and avoidance of indwelling lines and their associated complications [27].

MMC and 5FU are an alternative first line combination although there is less evidence to suggest an impact on survival. In a retrospective series, three of eight patients treated with MMC and 5FU had a response to treatment in terms of decrease in tumour size, reduction in pain, and improvement in performance status [28].

In an early phase II study, vincristine, bleomycin, and high-dose methotrexate were given to 15 patients; three out of the twelve patients with measurable disease (25%) had objective responses of 1-, 2-, and 5-month duration. However five of the fifteen patients had severe or life-threatening complications as a result of this treatment regimen and subsequently the regimen was deemed too toxic to warrant further evaluation [13].

In a single arm trial of twenty patients treated with mitomycin C, adriamycin, cisplatin, and bleomycin-CCNU, 12 (60%) patients had a partial response and no complete responses were observed. The median survival in this study was 15 months (95% CI 6–20 months), and the median time to progression or death was 8 months (95% CI 4–9 months). Toxicity was not insignificant, and although these side effects were managed without sequelae, the combination has not been explored in a phase III trial due to toxicity [15].

The most encouraging results have been demonstrated in a phase II single arm study of paclitaxel, carboplatin, and continuous infusional 5FU in patients with advanced squamous carcinomas (*n* = 60) from multiple primary sites, given first or second line. Seven patients with SCC of the anus were included, of these four responded, two of whom had a complete response. The patients with a complete response then received consolidation radiotherapy to the primary tumour. The median duration of response in the anal subgroup was 26 months (range 10–63+) [14]. Although most patients were able to tolerate full doses of all three drugs, gastrointestinal toxicities (mucositis and diarrhoea) were responsible for most dose reductions. Interestingly substantial activity of this three-drug combination was demonstrated across all tumour types in the trial, with an overall complete response rate of 25%.

There have been several single case reports or series evaluating alternative monotherapy or combination chemotherapy regimens. These data are limited by relatively small numbers and lack of prospective validation. The three-drug regimen, paclitaxel, ifosfamide and cisplatin (TIP), has been used in head and neck cancer and cervical squamous cell carcinomas, and a recent case series reported three patients with metastatic anal SCC treated with TIP. All three patients had a complete response with disease-free intervals of 4, 6, and 36 months, all required prophylactic GCSF support during their treatment [19]. 

## 3. Localised Therapies

The liver is the most common site of metastases, and the role of regional therapy has been explored in a multicentre analysis of 52 patients with squamous cell carcinomas, 27 of whom had metastatic anal cancer. Patients underwent hepatic resection (*n* = 47), radiofrequency ablation (*n* = 3), or both (*n* = 2), and the majority had received systemic therapy prior to resection. When the analysis was restricted to the 27 patients with metastatic anal cancer, the median disease-free and overall survival durations were 9.6 and 22.3 months, respectively [32]. These data suggest that there may be a subset of patients with isolated hepatic metastases who may benefit from resection, but selection criteria are as yet undefined and metastasectomy is not currently standard practice.

Palliative surgery for the primary lesion in those with metastatic disease and symptomatic local disease is an option. For those with significant comorbidities unable to tolerate systemic chemotherapy or surgery palliative radiotherapy alone is an alternative local therapy. Effective palliation can be achieved with a dose of 45–54 Gy in 25–30 fractions; however, alternative shorter or hypofractionated regimens such as 30 Gy in 10 fractions or 6 Gy/week for 5-6 weeks may be more appropriate for frailer patients.

The role of a shortened course of chemoradiation (30 Gy in 10 fractions with concurrent 5FU) has also been evaluated in elderly patients, demonstrating high rates of local control [33].

## 4. The Role of EGFR-Targeted Therapies in Anal Cancer

Squamous cell carcinoma of the anal canal has a number of similarities to cervical and head and neck squamous tumours, in terms of histology, a strong causative association with HPV infection and relative radiosensitivity. Squamous cell carcinoma of the anus, in common with cervical and head and neck squamous tumours, regularly overexpresses the epidermal growth factor receptor (EGFR). In a large cohort of invasive anal SCC tissue samples (*n* = 101) immunohistochemistry analysis of EGFR demonstrated that the majority of invasive anal SCCs overexpressed EGFR. Of the 101 patient biopsies available, 82 samples had sufficient material for interpretation of these 72/82 (90%) stained positive for EGFR, while 41/82 (50%) samples displayed at least moderate to strong staining [34]. Similar results have been demonstrated in a number of smaller studies, with range of EGFR overexpression of 55–100% [35, 36]. Interestingly EGFR gene amplification was not observed in these studies suggesting that overexpression occurs via mechanisms other than gene amplification.

There are data to suggest a correlation between oncogenic HPV and EGFR expression. In a study by Walker et al. 96% of invasive HPV-infected anal SCCs displayed strong membrane immunoreactivity to EGFR expression. It is also thought that HIV-positive status contributes in augmenting EGFR expression levels that are involved in carcinogenesis [37].

Mutations in the downstream effectors *KRAS* and *BRAF*, which are responsible for a lack of response to anti-EGFR antibodies in colorectal adenocarcinomas, appear rarer in anal tumours [38]. In a study of 95 tumour biopsies from squamous cell anal cancers, the tumours lacked the common *KRAS *and EGFR mutations. Only three samples had an EGFR exon 21 mutation and none had *KRAS* mutations. In a smaller series of three tumours that expressed EGFR, none had a mutation in *KRAS* codon 12 or 13 [39]. The anti-EGFR monoclonal antibody cetuximab has demonstrated efficacy in head and neck squamous cell tumours when administered concomitantly with radiotherapy [40]. In a case series of 7 heavily pretreated anal cancer patients, cetuximab was given in combination with irinotecan. In contrast to previous studies, *KRAS *mutations were demonstrated in a number of patients in this case series. Response (defined as partial or minor) was seen in the* KRAS* wild type patients (*n* = 5) with no response demonstrated in the patients with *KRAS* mutations (*n* = 2) [30]. These findings correlate with the results of two case reports of patients who achieved an excellent response to treatment with irinotecan and cetuximab having previously failed irinotecan [29, 31] (Table 3).

These data in a relatively small patient population and the presence of EGFR overexpression in anal SCC suggest that the combination of irinotecan and cetuximab warrants further evaluation. An alternative combination of paclitaxel with cetuximab has been evaluated in a number of squamous cell carcinomas with encouraging results. In a phase II trial of patients with stage IV non-small-cell lung cancer (NSCLC), a 3-weekly combination of paclitaxel (100 mg/m^2^/week for 3 weeks), carboplatin (area under curve = 6), and cetuximab (400 mg/m^2^ loading dose followed by 250 mg/m^2^/week) was safe and well tolerated; the response rate was 57% with an overall survival of 13.8 months [41]. A weekly regimen of paclitaxel (80 mg/m^2^/week) with cetuximab (400 mg/m^2^ loading dose followed by 250 mg/m^2^/week) was evaluated in a phase II study in squamous cell carcinoma of the head and neck; the combination was well tolerated with an overall response rate of 71% and disease control rate of 88% [42]. In light of similarities in the histology and pathogenesis between squamous cell carcinomas, these results suggest that the combination of paclitaxel and cetuximab is worthy of exploration in anal cancer.

## 5. Prognostic Factors

Poor prognostic factors include male sex, age over 65 years, more advanced T stage, nodal involvement, or poorly differentiated histology. In a review of prognostic factors derived from a prospective database male sex and clinically node positive disease were confirmed as poor prognostic factors for both disease-free and overall survival [43]. Black African Americans have worse outcomes than white American patients. Anal cancer is not an AIDS-defining illness; however, patients with HIV appear to have lower overall survival rates compared to HIV-negative patients [44].

A number of potential prognostic biomarkers have been evaluated in small studies; the tumour suppressor genes p53 and p21 are the only markers found to have an association with outcome in more than one study [45]. Even these results are not consistent; p53 was associated with worse outcome in two studies but no association was demonstrated in six others [46–48]. Variability in methodology and small patient numbers may explain the discordance results [49, 50]. Alternative potential markers including apoptotic regulators (Bcl2 BAX, and NF-KB), EGFR, cyclins A, D, and E, markers of proliferation (ki67, and MiBL), and angiogenic factors have also been evaluated in small studies. None of the results have been replicated in larger studies, and as yet there is no biomarker with the ability to predict outcome in this disease.

## 6. Conclusions

There have been very few developments in the treatment of metastatic disease for some time and currently there are no ongoing phase II or III trials specifically for anal cancer. In the era of Personalized medicine it would appear time for a shift in the treatment paradigm for patients with metastatic anal cancer. New experimental regimens may achieve better results and novel strategies of combination cytotoxic platforms with anti-EGFR targeted agent may lead to improved efficacy. In order to facilitate research and implement more effective treatment strategies clinical trials incorporating tissue collection for biomarkers are essential. International collaboration in global clinical trials is imperative and could improve outcomes in this setting.

## Figures and Tables

**Table 1:** Phase II chemotherapy trials of metastatic squamous cell carcinoma of the anus.

Author	Number of patients	Agents	Response rate	Median PFS (months)	OS (months)
Wilking et al. [13]	15	Vincristine, bleomycin, and high-dose methotrexate	3/12 (25%)	2	NR
Hainsworth et al. [14]	60 (7 with anal cancer)	Paclitaxel, carboplatin, and infusional 5FU	65% overall 4/7 (57%) anal patients	35 overall (26 anal patients)	NR
Jhawer et al. [15]	20	Mitomycin C, adriamycin, cisplatin, and bleomycin-CCNU	12/20 (60%)	8	15

**Table 2:** Case reports of single-agent chemotherapy in metastatic anal squamous cell carcinoma.

Author	Number of patients	Agent	Response	PFS/duration of response (months)	OS
Evans et al. [16]	1	Carboplatin	Partial	9	NR
Fisher et al. [17]	1	Doxorubicin and cis-dichlorodiammineplatinum	Major	NR	NR
Zimm and Wampler [18]	1	Semustine	Partial	15	NR
Golub et al. [19]	3	TIP	Complete (3/3)	4,6,36	NR
Grifalchi et al. [20]	1	Irinotecan	Partial	NR	NR

**Table 3:** Studies of cetuximab in metastatic anal squamous cell carcinoma.

Author	Number of patients	Agent	Response	PFS/duration of response	OS
De Dosso et al. [29]	1	Irinotecan + cetuximab	Partial	Patient died of PE on treatment	NR
Lukan et al. [30]	7	Irinotecan + cetuximab	5/7 response* (3 partial, 1 minor, and 1 stable)	6	NR
Phan and Hoff [31]	1	Irinotecan + cetuximab	Partial	NR	NR

* All five that responded were KRAS wild type.
